# Contemporary Diagnosis and Management of Patients with MINOCA

**DOI:** 10.1007/s11886-023-01874-x

**Published:** 2023-04-17

**Authors:** Purvi Parwani, Nicolas Kang, Mary Safaeipour, Mamas A. Mamas, Janet Wei, Martha Gulati, Srihari S. Naidu, Noel Bairey Merz

**Affiliations:** 1 Division of Cardiology, Department of Medicine, Loma Linda University Health, Loma Linda, CA USA; 2 Loma Linda University School of Medicine, Loma Linda, CA USA; 3 Keele Cardiovascular Research Group, Institute for Prognosis Research, University of Keele, Keele, UK; 4 Barbara Streisand Women’s Heart Center, Cedars-Sinai Smidt Heart Institute, Los Angeles, CA USA; 5 Department of Cardiology, Westchester Medical Center, Valhalla, NY USA

**Keywords:** MINOCA, INOCA, CMD, VSA, PET, Stress CMR, Microvascular disease

## Abstract

**Purpose of Review:**

Myocardial infarction with nonobstructive coronary arteries (MINOCA) is defined as acute myocardial infarction (MI) with angiographically no obstructive coronary artery disease or stenosis ≤ 50%. MINOCA is diagnostically challenging and complex, making it difficult to manage effectively. This condition accounts for 6–8% of all MI and poses an increased risk of morbidity and mortality after diagnosis. Prompt recognition and targeted management are essential to improve outcomes and our understanding of this condition, but this process is not yet standardized. This article offers a comprehensive review of MINOCA, delving deep into its unique clinical profile, invasive and noninvasive diagnostic strategies for evaluating MINOCA in light of the lack of widespread availability for comprehensive testing, and current evidence surrounding targeted therapies for patients with MINOCA.

**Recent Findings:**

MINOCA is not uncommon and requires comprehensive assessment using various imaging modalities to evaluate it further.

**Summary:**

MINOCA is a heterogenous working diagnosis that requires thoughtful approach to diagnose the underlying disease responsible for MINOCA further.

## Introduction

Acute myocardial infarction (MI) is diagnosed with acute myocardial injury and in the setting of myocardial ischemia [1]. While obstructive coronary artery disease (CAD) with underlying plaque disruption (type 1 MI) causes most MI, 6–8% of MI occur in the setting of nonobstructive coronary arteries (MINOCA) [1]. In practice, MINOCA cases can be challenging, as identifying the etiology and appropriately guiding therapy require additional diagnostic evaluations [2, 3••].

By the Fourth Universal Definition, MI is defined by myocardial injury (i.e., elevated cardiac troponin (cTn) above the 99th percentile) that is acute (i.e., rising or falling cTn) and ischemic (i.e., new ischemic symptoms, Q-waves on electrocardiogram, loss of viable myocardium, or regional wall motion abnormalities on imaging) [1]. Although angiographically nonobstructive coronaries (no stenosis ≥ 50%) after MI can raise suspicion for MINOCA, comprehensive diagnostic evaluation should exclude overt clinical causes of troponin elevation, inadvertently overlooked obstructive CAD, and nonischemic myocardial injury (i.e., myocarditis and takotsubo syndrome (TS)) before identifying an ischemic cause of myocardial injury [1]. In a large systemic review including 55,369 suspected MINOCA cases from 23 studies, MINOCA had unfavorable outcomes (all-cause mortality = 3.4%, cardiovascular mortality = 1.8%, composite MACE prevalence = 9.6%, reinfarction rate = 2.6%, heart failure hospitalization rate = 3.9%, and a stroke admission rate = 1.0% 12 month after diagnosis) most significantly during hospitalization and after 1 month [4•]. Hence, contrary to historic opinion, MINOCA carries a significant burden of disease a year after diagnosis, and thus, prompt recognition and treatment of these patients is essential  [1]. A summary of the defining features of MINOCA is seen in Table 1.*cTn* cardiac troponin; *FFR* fractional flow reserve; *MINOCA* myocardial infarction with no obstructive coronary arteries

**Table 1:** Definition of suspected MINOCA

**Defining features for MINOCA**	**Diagnostic criteria**
1. Acute myocardial infarction based on the Fourth Universal Definition of Myocardial Infarction	• Serial cTn values with a rise or fall with at least one value above the 99th percentile upper reference limit with new clinical evidence of infarction evidenced by the following: o Symptoms of myocardial ischemia o Ischemic changes or pathologic Q-waves on electrocardiogram (ECG) o Loss of viable myocardium or wall motion abnormalities on imaging o Coronary thrombus identified on angiography or autopsy
2. Absence of obstruction in any major epicardial artery based on angiographic guidelines	• Absence of lesion causing stenosis ≥ 50% in a major epicardial vessel without FFR • Absence of borderline stenosis (≥ 30 but < 50%) in a major epicardial vessel with FFR > 80%
3. Absence of clinically overt alternative cause for the acute presentation	• Operative stress • Sepsis • Arrhythmia • Heart failure • Anemia
4. Absence of nonischemic causes of myocardial injury	• Myocarditis • Takotsubo syndrome • Other cardiomyopathies

*Suspected MINOCA is defined by the presence of all four defining features (1–4)

At the time of angiography, MINOCA is considered a working diagnosis, until other causes of myocardial injury are excluded and the underlying ischemic mechanism is identified [1]. MINOCA is caused by a large and heterogenous group of etiologies (plaque disruption–plaque rupture, erosion, and calcified nodules; coronary microvascular dysfunction [CMD]; thromboembolism; coronary artery spasm; etc.) [1, 3••, 5]. Thus, a diagnostic approach that differentiates ischemic from nonischemic etiologies of myocardial injury and then further characterizes the ischemic mechanism can be practical for guiding management [3••]. This review will discuss the epidemiology and how invasive and noninvasive diagnostics can guide the management of MINOCA[3, 5, 6].

## MINOCA Prevalence and Demographic Characteristics

The clinical profile of MINOCA is unique and different from obstructive CAD. Pasupathy et al. [7••] meta-analyzed 28 studies and reported that MINOCA occurs in 6% of MI with a female prevalence of 40% diagnosed at a median age of 55. Also, MINOCA patients were more likely to be younger (55 vs. 61 years), female (43% vs. 24%), and had less hyperlipidemia (21% vs. 32%) than obstructive CAD, respectively. However, the prevalence of other traditional risk factors was similar between both groups, and hereditary thrombophilia was present in 14% of MINOCA patients. While most (66%) MINOCA present with NSTEMI and have better outcomes than obstructive CAD [7], it portends a guarded prognosis with a 12-month all-cause mortality of 3.4% [4].

## Initial Considerations for Suspected MINOCA

**Fig. 1:**
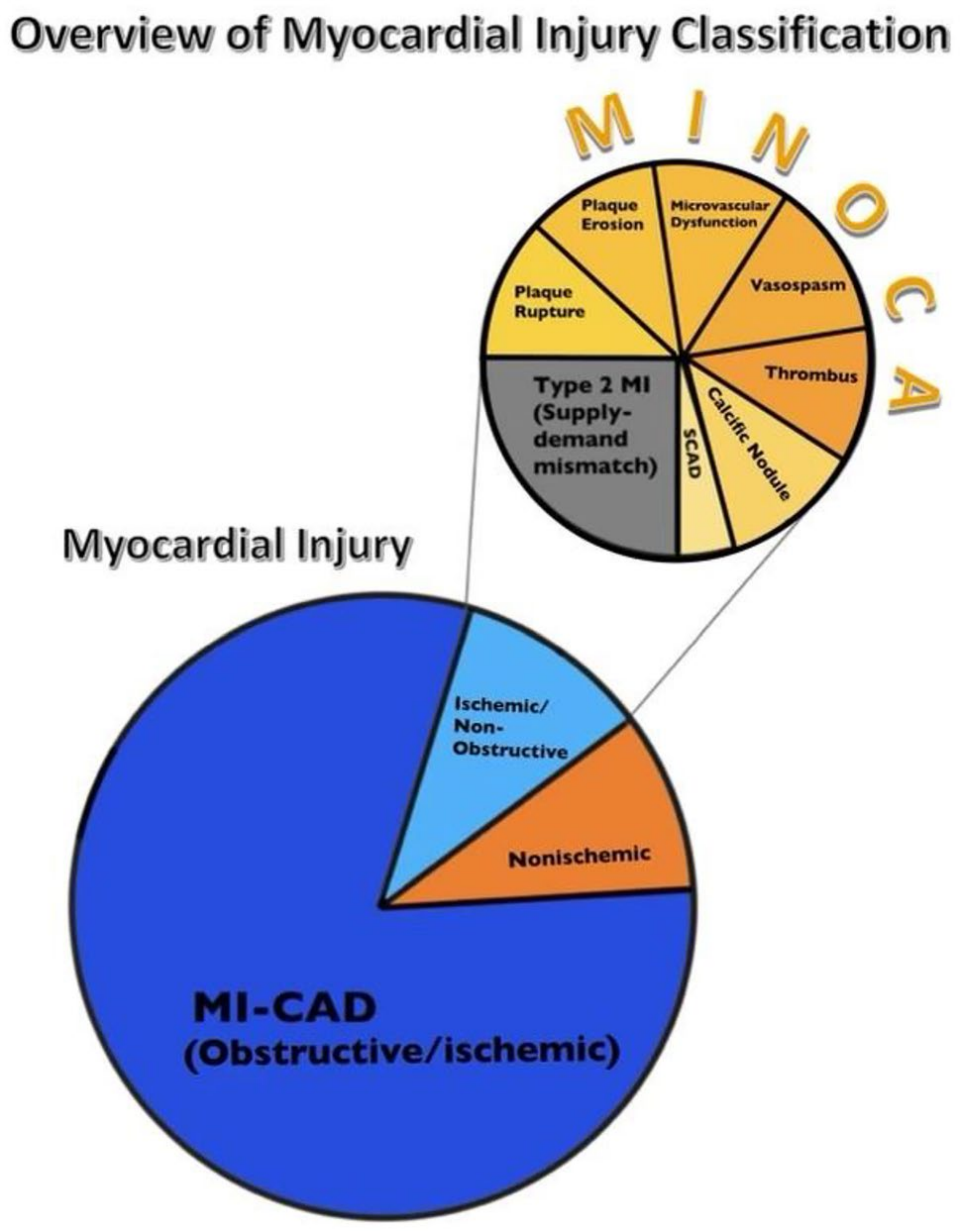
Myocardial injury classification. Abbreviations: MINOCA, myocardial infarction with nonobstructive coronary arteries; MI, myocardial infarction; MI-CAD, myocardial infarction due to obstructive coronary artery disease; SCAD, spontaneous coronary artery dissection. Proportions of ischemic and nonischemic myocardial injury based on Pasupathy et al. [9], significant variability with exact proportions of ischemic etiology of MINOCA and the figure merely has graphical representation of ischemic etiology of MINOCA

Initial evaluation of MINOCA includes assessing the clinical presentation and reviewing angiography findings in order to exclude clinically overt and systemic causes of type 2 MI (sepsis, pulmonary embolism, cardiac contusion and other noncardiac causes for elevations in cardiac troponin), as well as commonly overlooked coronary artery lesions (complete occlusion of small coronary artery branches by plaque disruption or coronary artery embolism, ≥ 50% distal stenosis in a major epicardial artery, or spontaneous coronary artery dissection (SCAD)) [3]. Fig. 1 shows an overview of classifying myocardial injury using the principles described above.

## Noninvasive Imaging Evaluation

Further careful evaluation of systolic function by imaging particularly cardiac magnetic resonance (CMR) imaging can help identify ischemic and nonischemic causes of myocyte injury (myocarditis, TS, and other cardiomyopathies) [3]. In a meta-analysis that used CMR studies as a diagnostic tool in patients presenting with an initial suspected diagnosis of MINOCA, myocarditis was found to be a leading etiology, and MINOCA was thus excluded in more than one fourth of the cases [8]. TS and other cardiomyopathies can also mimic MINOCA and should be excluded before considering ischemic etiologies of troponin release. Pasupathy et al. combined 26 studies with around 1500 patients with an initial suspected MINOCA diagnosis, and 33% of patients had myocarditis, 18% had TS, and 12% had other cardiomyopathy diagnoses. Only 24% of the initial suspected diagnosis of MINOCA had evidence of MI on CMR. No diagnosis was established in around 26% of the patients [9].

Imaging with CMR is a critical step in the evaluation of suspected MINOCA. CMR is a safe, non-ionizing radiation test that assesses myocardial perfusion, ventricular function, and the underlying mechanism of myocardial injury while differentiating ischemic and nonischemic myocardial injury using T2 and late gadolinium enhancement (LGE) sequences. An ischemic injury appears along vascular territory as myocardial edema or fibrosis affecting the subendocardial or transmural myocardium on T2 and LGE sequences [10]; in contrast, a nonischemic injury shows LGE that affects epicardial or mid-myocardial regions and does not follow vascular territories [10]. Use of CMR within a week of index event results in higher diagnostic accuracy of around ~ 90% in evaluation of ischemic versus nonischemic injury; however, reasonable diagnostic accuracy up to ~ 70–80% is still achievable when CMR is done within 3 months of index event [11].

Coronary computed tomography angiography (CCTA) can be considered when cardiac catheterization, CMR, and intravascular imaging are unavailable or contraindicated, but evidence supporting its use in MINOCA is lacking [12]. CCTA can be useful to identify perfusion defects that suggest ischemia but may also detect high-risk plaque features, evidence of inflammation (ref: PMID: 31,462,127) and identify SCAD [13–15]. Although the Very Early Versus Deferred Invasive Evaluation Using Computerized Tomography in Patients with Acute Coronary Syndromes (VERDICT) trial [16] showed CCTA was effective ruling out obstructive CAD, the Myocardial Infarction With Non-Obstructive Coronary Arteries in the Greek Population (MINOCA-GR) trial [16] will be the first large observational study to study its utility in MINOCA patients.

In patients with suspected CMD as the mechanism of MINOCA, a reduced coronary flow reserve (CFR) < 2 or the ratio of coronary blood flow between maximal hyperemia and rest can be obtained noninvasively using transthoracic Doppler, stress CMR, or stress positron emission tomography (PET) [17, 18]. This is accomplished by measuring myocardial blood flow using PET [19], myocardial perfusion using CMR [20], and coronary flow velocity using pulsed wave transthoracic Doppler echocardiography [21], before and after vasodilator stress.

## Invasive Imaging Evaluation

Intracoronary vascular imaging with optical coherence tomography (OCT) and intravascular ultrasound (IVUS) can help evaluate coronary lesions that were not apparent on angiography [22]. Although large thrombi can cause obvious pruning on angiography, stenosis from plaque disruption, distal coronary artery embolization, and SCAD can be poorly visualized angiographically [23–25]. OCT can visualize luminal and superficial coronary artery lesions with high resolution and assess morphologic features at the tissue level; this makes OCT an ideal test to detect most lesions in MINOCA, including plaque disruption and its sequelae [26, 27] Furthermore, OCT or IVUS can be utilized for SCAD evaluation [28, 29].

A recent meta-analysis suggests the combination of intracoronary OCT followed by early CMR had a superior diagnostic yield than CMR alone [30]. Combined OCT then early CMR within 1 week of MI presentation identified a diagnosis of MINOCA or alternative, nonischemic causes of myocardial injury in 85–100% of patients with a working diagnosis of MINOCA, while CMR alone had a diagnostic yield of around 74%. The pooled incidence of nonischemic myocarditis (29%) and TS (12%) among suspected MINOCA was very high, but ischemic causes of MINOCA detected by OCT, such as plaque disruption (9–39%), plaque rupture (21–35%), plaque erosion (11–30%), lone thrombus (7.5–18%), calcific nodule (2.5–5%), and SCAD (0.7–5%), were less common [30]. These findings underscore the importance of early CMR when evaluating suspected MINOCA to exclude nonischemic myocardial injury, which is quite common in these patients. Provocative testing can also be obtained if microvascular or epicardial coronary vasospasm is suspected [22, 31].

Role of fractional flow reserve (FFR) measurement during MINOCA has not been yet studied systemically in patients with MINOCA; however [3], with recent evidence of use of FFR in patients with nonobstructive CAD and higher proportion of functionally significant coronary lesions, FFR use is preferred [32].

## Diagnostic Evaluation of Type 1 (Atherosclerotic) Acute Myocardial Infarction

Evaluation of coronaries with intravascular imaging can help classify MINOCA into type 1 MI (atherosclerotic) and type 2 MI (non-atherosclerotic) by readily identifying atherosclerotic causes particularly plaque disruption and its sequelae, namely, plaque rupture, erosion, and rarely calcified nodules by using intracoronary optical coherence tomography (OCT) [33••].

Plaque disruption may appear as a hazy or small filling defect on angiography but equally may not be evident. Intracoronary imaging with OCT or IVUS is required for a definitive diagnosis [1]. On OCT, plaque rupture is a discontinuous fibrous cap that communicates between the plaque cavity and the coronary lumen. In contrast, plaque erosion is a thrombus contiguous to a plaque’s luminal surface without signs of rupture [34].

## Diagnostic Evaluation of Type 2 (Non-atherosclerotic) Acute Myocardial Infarction

### Systemic Supply–Demand Mismatch

The fourth universal definition of myocardial infarction regards type 2 MI as ischemic events secondary to a supply–demand imbalance [1]. Evaluation should include close examination for plausible systemic insults, notably arrhythmias, surgery, infection, heart failure, pulmonary embolism, anemia, etc [35]. By the Fourth Universal Definition, a diagnosis of type 2 MI can be made with MI and a probable clinical insult without clinical, angiographic, or other invasive evidence to support an alternative diagnosis [6, 36]. The definitive diagnosis of a systemic cause of type 2 MI should then exclude further evaluation of a cause of MINOCA.

### Spontaneous Coronary Artery Dissection (SCAD)

SCAD is a commonly overlooked cause of MINOCA that requires a high index of suspicion, especially in young women with MI [3••]. There are three types of SCADs based on angiographic appearance [37]. Type 1 has contrast stains in the arterial wall with multiple radiolucent lumens with or without slow contrast clearing [37]. Type 2 shows diffuse, smooth, usually, 20–30 mm narrowing with varying severity, and type 3 shows focal or tubular stenosis that mimics atherosclerosis [37]. Both types 2 and 3 may require further evaluation with intravascular imaging with OCT or IVUS to confirm the presence of an intramural hematoma or false lumen if not clearly visualized on angiography [37]. In the absence of type 1 SCAD, Saw et al. suggest evaluating type 2 SCAD with intracoronary nitroglycerin and then OCT or IVUS if stenosis persists [28].

### Coronary Microvascular Dysfunction (CMD)

CMD is caused by a supply–demand mismatch that causes hypoperfusion most predominantly during hyperemic states and results from increased microvascular resistance, vasoreactivity, and impaired vasodilation. CMD is diagnosed with a CFR < 2 after vasodilator (adenosine) administration, an index of microvascular resistance (IMR) > 25, or a corrected TIMI frame count ≥ 3 beats to fill a vessel [18, 38, 39]. Although CMD is diagnosed definitively by invasive coronary functional testing, diagnosis can be made alternatively using noninvasive testing, e.g., transthoracic Doppler, stress CMR, or PET [17, 18]. Decreased coronary blood flow is evidenced by a corrected TIMI flow count demonstrating the slow flow phenomenon, or angiography showing contrast filling a vessel is delayed [39]. CFR by intracoronary Doppler and alternatively by thermodilution measures endothelium-independent vasodilation [40]. A reduced CFR indicates inability of the vasculature to vasodilate and increase coronary blood flow to meet metabolic demands during hyperemic states [40].

Intracoronary adenosine is well tolerated in patients with MI with PCI [41], but the diagnostic utility in MINOCA is unclear. Because CMD can be both a cause of MINOCA and a complication of myocardial injury, abnormal microvascular testing may not fully explain the etiology of MINOCA. Additionally, a positive test cannot distinguish ischemic from nonischemic insults, as CMD can occur even with nonischemic myocardial injury, such as myocarditis [42]. This further reinforces why CMR is useful to exclude nonischemic injury in suspected MINOCA before additional testing is considered [42]. Hence, while the coronary microvascular assessment at the time of provocative testing appears safe and feasible, testing availability and data regarding the usefulness of coronary microvascular testing in MINOCA is limited. However, persistent symptoms after index presentation of MINOCA should prompt further evaluation of INOCA.

### Coronary Vasospasm

Provocative coronary functional testing can evaluate vasospasm due to suspected coronary microvascular dysfunction (CMD) and epicardial coronary vasospasm [31]. Acetylcholine infusion with epicardial vasospasm visualization on coronary angiography is the reference gold standard test to diagnose coronary epicardial vasospasm [43–45]. Ong et al. reported in the CASPAR study that up to 50% of patients with MINOCA had coronary vasospasm after acetylcholine infusion, [46] and many studies suggest this test is well tolerated in MI [32, 44, 47]. A meta-analysis with 71,566 patients with ischemia with nonobstructive coronary arteries (INOCA) reported the incidence of complications after acetylcholine infusion to be 0.5%, with no reported deaths [47]. Complications included ventricular fibrillation or tachycardia in 0.2%, atrial fibrillation in 0.1%, transient bradycardia or advanced atrioventricular block in 0.1%, and prolonged refractory spasm without MI in 0.1% [43]. Additionally, the incidences of arrhythmias with acetylcholine in MINOCA, INOCA, and after spontaneous vasospasms were similar [44, 47].

Several studies suggest that provocative testing during index hospitalization or by referral to capable facilities can be beneficial for the following reasons. First, a positive provocative test in MINOCA is safe, and arrhythmias are rare [43, 47, 48]. Second, it can further help identify MINOCA patients with poor outcomes [32, 47]. Last but most importantly, by making a diagnosis, it improves rates of appropriate medical therapy with calcium channel blockers (CCB) on discharge and other effective antianginals for vasospasm [32].

Epicardial vasospasm and microvascular vasospasm contributors are endothelial dysfunction at the arterial and arteriolar levels, respectively [49]. Endothelial dysfunction commonly coexists at both levels and is assessed by provocative testing using intracoronary acetylcholine, which stimulates coronary vasoconstriction and reproduces ischemic symptoms [49]. By the Coronary Artery Vasospastic Disorders Summit (COVADIS) criteria [50], definitive vasospastic angina is diagnosed with a nitrate-responsive angina (e.g., during a spontaneous episode and one of the following — rest angina, morning exercise intolerance, hyperventilation-triggering, or improvement with CCB but not β-blockers) with either transient ischemic electrocardiographic changes (e.g., ST elevation or depression ≥ 0.1 mV, new negative U-wave) or coronary artery spasm (e.g., > 90% coronary artery constriction with angina and ischemic electrocardiographic changes either spontaneously or with acetylcholine, ergot, or hyperventilation).

### Coronary Artery Embolism

Coronary artery embolism is an uncommon cause of MINOCA and requires a high index of suspicion based on history and laboratory testing [3••]. Coronary artery embolism can result from extra-coronary thromboembolism or as a complication of plaque disruption [3••]. Clinicians should consider coronary artery embolism in patients with MI and risk factors for thromboembolism, especially atrial fibrillation, prosthetic valves, atrial septal defect, left-sided valvular disease (infectious endocarditis, aortic or mitral calcification), intracardiac tumors, and thrombophilia (factor V Leiden, protein C and protein S deficiency, factor XII deficiency, malignancy, systemic lupus erythematous) [51, 52]. If valvular dysfunction is the suspected cause, transesophageal echocardiogram should be obtained to assess valvular function and the presence of embolic foci (vegetations and tumors) [51–53].

**Fig. 2:**
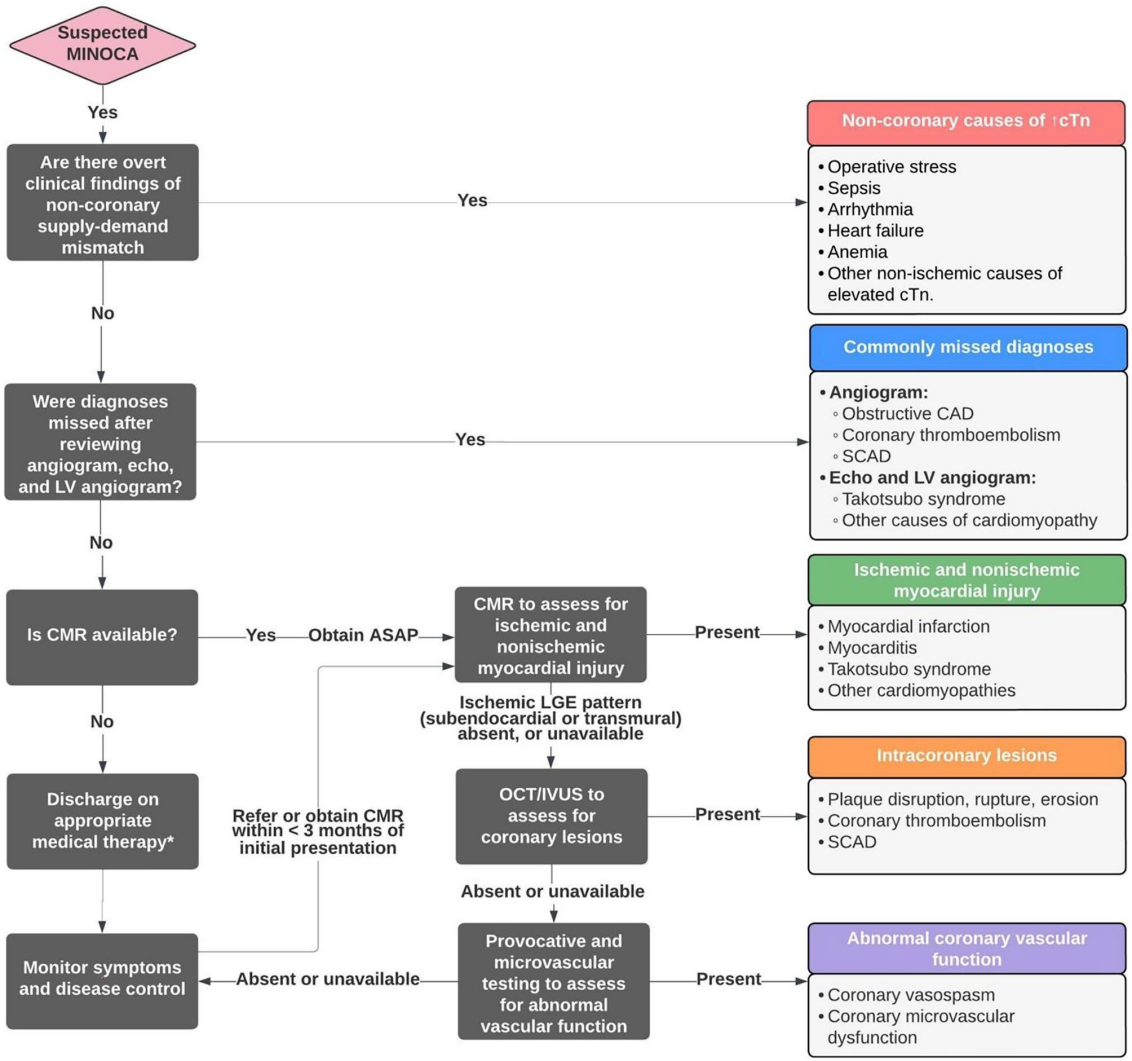
Diagnostic algorithm for evaluating patients with suspected MINOCA. Abbreviations: CMR, cardiac magnetic resonance; cTn, cardiac troponin; LV, left ventricular; MINOCA, myocardial infarction with nonobstructive coronary arteries; TEE, transesophageal echocardiogram; VF, ventricular fibrillation; VT, ventricular tachycardia

Figure 2 summarizes the diagnostic strategies for evaluating patients with a working diagnosis of MINOCA, as discussed previously.

## The General Approach to Management of Patients with MINOCA

Patients diagnosed with an underlying cause for MINOCA benefit from cause-directed treatment, while secondary atherothrombotic prevention measures should be considered depending on the etiology [1, 5]. Currently, consensus recommendations from guidelines are absent. For example, ESC recommends routine use of aspirin, statins, and CCB for vasospasm [3••]. However, AHA reserves statins and antiplatelet use for MINOCA caused by plaque disruption and recommends avoiding in type 2 MI, as it may be contraindicated [1]. Some studies suggest a long-term benefit through cardiac rehabilitation, ACEI, and ARB [54, 55]. Although the MINOCA-BAT (Randomized Evaluation of Beta Blocker and ACEI/ARB Treatment in MINOCA Patients) [56•] and WARRIOR (Women’s Ischemia Trial to Reduce Events In Non-Obstructive CAD) [57] trials are in progress, current guideline-based recommendations are mixed, and selecting therapy should target the underlying etiology [1, 5].

### Plaque Disruption

Currently, secondary prevention with aspirin, statin, ACEI or ARB, lifestyle modification, and cardiac rehabilitation is mainly designed to treat type 1 MI, which may similarly be beneficial in MINOCA patients with an etiology of plaque disruption [58]. The addition of a β-blocker and P2Y12 inhibitor (i.e., clopidogrel), however, remains controversial. Although most studies show no additional benefit with the addition of a β-blocker and P2Y12 inhibitor in patients with MINOCA, treatment effects were analyzed in the entire MINOCA cohort without differentiating the effects in each etiology of MINOCA. Thus, prospective trials evaluating the efficacy of these treatments in MINOCA patients with underlying plaque disruption are necessary to guide management of these patients [54].

### Coronary Artery Vasospasm

Concurrent use of both short-acting nitrates and CCB (dihydropyridine and non-dihydropyridine) is effective for treating active spasms, preventing recurrent symptoms and arrhythmias, and improving mortality. When angina is refractory to short-acting nitrates, long-acting nitrates can provide symptomatic relief. Although the clinical benefit of short-acting nitrates is well-defined, the efficacy of long-acting nitrates is unclear possibly due to tolerance [59, 60]. Low-dose aspirin is also effective in treating coronary vasospasm by inhibiting thromboxane-A2-mediated-vasoconstriction, but large doses may worsen vasospasms through prostacyclin inhibition and should be used with caution. Furthermore, the addition of statins, cilostazol, and nicorandil (an ATP-sensitive potassium channel modulator with nitrate-like properties) may also be beneficial in patients with vasospasm [61].

### Coronary Microvascular Dysfunction

The evidence necessary to appropriately guide the management of MINOCA due to CMD is scarce. While studies suggest treatment with β-blockers, long-term L-arginine, dipyridamole, and ranolazine can provide symptomatic relief, studies that report functional improvement with these therapies are limited to CMD patients with stable angina [61–63]. Furthermore, while the evidence supporting ACEI or ARB as an effective monotherapy for MINOCA due to CMD is weak, several studies suggest that combined use of an aldosterone antagonist with an ACEI or ARB may offer additional clinical benefit in CMD [64].

### Spontaneous Coronary Artery Dissection

There are no randomized clinical trials that compare the efficacies of medical or revascularization therapies for SCAD. Saw et al. suggests initially identifying clinical features of revascularization candidates angiographically (left main dissection) or clinically (ongoing or recurrent chest pain or ischemia, ventricular arrhythmias, and cardiogenic shock). Patients with high-risk clinical features for revascularization and hemodynamic instability should be considered for advanced cardiac implantable devices, including intra-aortic balloon pump, extracorporeal membrane oxygenation, left ventricular assist device, and implantable cardioverter-defibrillator. Percutaneous coronary intervention (PCI) is then considered for isolated left main SCAD or after hemodynamic instability is stabilized. Coronary artery bypass graft, however, should be reserved for stable patients with (1) high-risk clinical features and either an ostial left anterior descending artery (LAD) lesion or ≥ 2 proximal lesions when PCI is not feasible or (2) left main dissection involving the LAD or left circumflex artery [65].

Fortunately, observational studies report 70% of SCAD resolve on repeat angiography, suggesting conservative medical therapy and inpatient monitoring are sufficient for most cases [28, 66, 67]. Experts and MI guidelines support routine use of low-dose aspirin, β-blockers, and either ACEi or ARB if left ventricular systolic dysfunction is present once reproductive-aged women are counseled about potential teratogenicity with ACEi and ARB [28, 29]. They also support regular exercise [68] and cardiac rehabilitation [69] as beneficial and safe therapies for MINOCA patients with SCAD; in fact, no evidence reports harm through heavier exercise loads [68]. While experts debate whether 1-year or lifelong aspirin therapy is more effective, DAPT without recent PCI should be avoided as hematoma and dissection can expand.

### Coronary Artery Embolism

Treatment of coronary thromboembolic conditions should focus on managing the underlying cause. There are currently no prospective, randomized control trials recommending either long-term anticoagulation or antiplatelet therapies to treat MINOCA due to coronary artery embolism. In the presence of a hematologic etiology, including thrombotic thrombocytopenic purpura, hematologic consultation should be considered [36].

## Conclusions

MINOCA is a heterogenous clinical condition that warrants further diagnostic evaluation to identify the underlying mechanism of myocardial infarction without significant obstructive coronary artery disease. A comprehensive and meticulous approach to diagnosis and determining the underlying etiology can further help risk stratify these patients and manage them more effectively.

## Abbreviations

ACEi: Angiotensin-converting enzyme inhibitor
ACS: Acute coronary syndrome
ARB: Angiotensin receptor blockers
CAD: Coronary artery disease
CCB: Calcium channel blockers
CCTA: Coronary computed tomography angiography
CFR: Coronary flow reserve
CMD: Coronary microvascular dysfunction
CMR: Cardiac magnetic resonance
CTA: Computed tomographic angiography
DAPT: Dual antiplatelet therapy
IMR: Index of microvascular resistance
INOCA: Ischemia with nonobstructive coronary arteries
IVUS: Intravascular ultrasound
LAD: Left anterior descending artery
MACE: Major adverse cardiovascular events
MINOCA: Myocardial infarction with nonobstructive coronary arteries
OCT: Optical coherence tomography
PET: Positron emission tomographic imaging
VSA: Vasospastic angina

## References

[1] Thygesen K, Alpert JS, Jaffe AS (2018). Fourth universal definition of myocardial infarction (2018). Circulation.

[2] Davies MJ (2000). The pathophysiology of acute coronary syndromes. Heart.

[3] •• Tamis-Holland JE, Jneid H, Reynolds HR, et al. Contemporary diagnosis and management of patients with myocardial infarction in the absence of obstructive coronary artery disease: a scientific statement from the American Heart Association. Circulation. 2019;139(18):E891-E908. 10.1161/CIR.0000000000000670/FORMAT/EPUB. **This study is important because it was the first scientific statement from AHA that described the diagnostic approach to patients presenting with MINOCA.** 10.1161/CIR.0000000000000670 30913893

[4] • Pasupathy S, Lindahl B, Litwin P, et al. Survival in patients with suspected myocardial infarction with nonobstructive coronary arteries: a comprehensive systematic review and meta-analysis from the MINOCA global collaboration. Circ Cardiovasc Qual Outcomes. 2021;14(11):e007880. 10.1161/CIRCOUTCOMES.121.007880/FORMAT/EPUB. **This study is important because it shows that MINOCA, a condition previously dismissed as benign, has increased risk of adverse outcomes compared to healthy subjects but had a favorable prognosis compared to MI-CAD.** 10.1161/CIRCOUTCOMES.121.007880 34784229

[5] Agewall S, Beltrame JF, Reynolds HR, et al. Current opinion ESC working group position paper on myocardial infarction with non-obstructive coronary arteries on behalf of the WG on Cardiovascular Pharmacotherapy. 10.1093/eurheartj/ehw149.

[6] Thygesen K, Alpert JS, Jaffe AS (2018). Fourth universal definition of myocardial infarction (2018). Circulation.

[7] •• Pasupathy S, Air T, Dreyer RP, Tavella R, Beltrame JF. Systematic review of patients presenting with suspected myocardial infarction and nonobstructive coronary arteries. Circulation. 2015;131(10):861–870. 10.1161/CIRCULATIONAHA.114.011201/FORMAT/EPUB. **This meta-analysis was the first one that demonstrated MINOCA should be considered as a working diagnosis with multiple potential causes that require evaluation so that directed therapies may improve its guarded prognosis.** 10.1161/CIRCULATIONAHA.114.011201 25587100

[8] Hausvater A, Smilowitz NR, Li B (2020). Myocarditis in relation to angiographic findings in patients with provisional diagnoses of MINOCA. JACC Cardiovasc Imaging.

[9] Pasupathy S, Tavella R, Beltrame JF (2017). Myocardial infarction with nonobstructive coronary arteries (MINOCA): the past, present, and future management. Circulation.

[10] Ferreira VM, Schulz-Menger J, Holmvang G (2018). Cardiovascular magnetic resonance in nonischemic myocardial inflammation: expert recommendations. J Am Coll Cardiol.

[11] Dastidar AG, Baritussio A, de Garate E (2019). Prognostic role of CMR and conventional risk factors in myocardial infarction with nonobstructed coronary arteries. JACC Cardiovasc Imaging.

[12] Gudenkauf B, Hays AG, Tamis-Holland J (2022). Role of multimodality imaging in the assessment of myocardial infarction with nonobstructive coronary arteries: beyond conventional coronary angiography. J Am Heart Assoc.

[13] Gonzalez JA, Lipinski MJ, Flors L, Shaw PW, Kramer CM, Salerno M (2015). Metaanalysis of diagnostic performance of computed coronary tomography angiography, computed tomography perfusion and computed tomography-fractional flow reserve in functional myocardial ischemia assessment versus invasive fractional flow reserve. Am J Cardiol.

[14] Oikonomou EK, Marwan M, Desai MY (2018). Non-invasive detection of coronary inflammation using computed tomography and prediction of residual cardiovascular risk (the CRISP CT study): a post-hoc analysis of prospective outcome data. The Lancet.

[15] Tweet MS, Gulati R, Williamson EE, Vrtiska TJ, Hayes SN (2016). Multimodality imaging for spontaneous coronary artery dissection in women. JACC Cardiovasc Imaging.

[16] Rampidis GP, Kampaktsis PN, Kouskouras K, et al. Role of cardiac CT in the diagnostic evaluation and risk stratification of patients with myocardial infarction and non-obstructive coronary arteries (MINOCA): rationale and design of the MINOCA-GR study. BMJ Open. 2022;12(2). 10.1136/BMJOPEN-2021-054698. 10.1136/bmjopen-2021-054698 PMC8811605 35110321

[17] Noel C, Merz B, Pepine CJ, Walsh MN, Fleg JL, Vincent S. Ischemia and no obstructive coronary artery disease (INOCA): developing evidence-based therapies and research agenda for the next decade. 10.1161/CIRCULATIONAHA.116.024534. 10.1161/CIRCULATIONAHA.116.024534 PMC5385930 28289007

[18] Ong P, Camici PG, Beltrame JF (2017). International standardization of diagnostic criteria for microvascular angina ☆. Published online.

[19] Murthy VL, Naya M, Taqueti VR, et al. Effects of gender on coronary microvascular dysfunction and cardiac outcomes. 10.1161/CIRCULATIONAHA.113.008507.

[20] Thomson LEJ, Wei J, Agarwal M, et al. Cardiac magnetic resonance myocardial perfusion reserve index is reduced in women with coronary microvascular dysfunction: a national heart, lung and blood institute-sponsored study from the women’s ischemia syndrome evaluation (WISE). 10.1161/CIRCIMAGING.114.002481. 10.1161/CIRCIMAGING.114.002481 PMC4375783 25801710

[21] Galiuto L, Sestito A, Barchetta S (2007). Noninvasive evaluation of flow reserve in the left anterior descending coronary artery in patients with cardiac syndrome X. Am J Cardiol.

[22] Collet JP, Thiele H, Barbato E, et al. 2020 ESC Guidelines for the management of acute coronary syndromes in patients presenting without persistent ST-segment elevation. The task force for the management of acute coronary syndromes in patients presenting without persistent ST-segment elevation of the European Society of Cardiology (ESC). Eur Heart J. 2021;42(14):1289–1367. 10.1093/EURHEARTJ/EHAA575. 10.1093/eurheartj/ehaa575 32860058

[23] Stone GW, Maehara A, Lansky AJ, et al. A prospective natural-history study of coronary atherosclerosis Abstract. N Engl J Med. 2011;364:226–261. 10.1056/NEJMoa1002358. 10.1056/NEJMoa1002358 21247313

[24] Xing L, Higuma T, Wang Z (2017). Clinical significance of lipid-rich plaque detected by optical coherence tomography: a 4-year follow-up study. J Am Coll Cardiol.

[25] Alfonso F, Paulo M, Dutary J (2012). Endovascular imaging of angiographically invisible spontaneous coronary artery dissection. JACC Cardiovasc Interv.

[26] Yabushita H, Bouma BE, Houser SL (2002). Characterization of human atherosclerosis by optical coherence tomography. Circulation.

[27] Jang IK, Tearney GJ, MacNeill B (2005). In vivo characterization of coronary atherosclerotic plaque by use of optical coherence tomography. Circulation.

[28] Saw J, Mancini GBJ, Humphries KH (2016). Contemporary review on spontaneous coronary artery dissection. J Am Coll Cardiol.

[29] Gilhofer TS, Saw J (2019). Spontaneous coronary artery dissection: a review of complications and management strategies. Expert Rev Cardiovasc Ther.

[30] Machanahalli Balakrishna A, Ismayl M, Thandra A (2022). Diagnostic value of cardiac magnetic resonance imaging and intracoronary optical coherence tomography in patients with a working diagnosis of myocardial infarction with non-obstructive coronary arteries – a systematic review and meta-analysis. Curr Probl Cardiol Published online.

[31] Sörensson P, Ekenbäck C, Lundin M (2021). Early comprehensive cardiovascular magnetic resonance imaging in patients with myocardial infarction with nonobstructive coronary arteries. JACC Cardiovasc Imaging.

[32] Montone RA, Niccoli G, Fracassi F (2018). Patients with acute myocardial infarction and non-obstructive coronary arteries: safety and prognostic relevance of invasive coronary provocative tests. Eur Heart J.

[33] Reynolds HR, Maehara A, Kwong RY (2021). Coronary optical coherence tomography and cardiac magnetic resonance imaging to determine underlying causes of myocardial infarction with nonobstructive coronary arteries in women. Circulation.

[34] Vergallo R, Ren X, Yonetsu T (2014). Pancoronary plaque vulnerability in patients with acute coronary syndrome and ruptured culprit plaque: a 3-vessel optical coherence tomography study. Am Heart J.

[35] DeFilippis AP, Chapman AR, Mills NL (2019). Assessment and treatment of patients with type 2 myocardial infarction and acute nonischemic myocardial injury. Circulation.

[36] Tamis-Holland JE, Jneid H, Reynolds HR (2019). Contemporary diagnosis and management of patients with myocardial infarction in the absence of obstructive coronary artery disease: a scientific statement from the American Heart Association. Circulation.

[37] Saw J, Humphries K, Aymong E, et al. Spontaneous coronary artery dissection clinical outcomes and risk of recurrence. 2017. 10.1016/j.jacc.2017.06.053 28838364

[38] Gilard M, Hasdai D, Hatala R, et al. European Association of Cardiovascular Imaging (EACVI), European Association of Preventive Cardiology (EAPC), European Association of Percutaneous Cardiovascular Interventions (EAPCI). Eur Heart Rhythm Assoc (EHRA). 10.1093/eurheartj/ehz425.

[39] Beltrame JF, Limaye SB, Horowitz JD, Beltrame JF. The coronary slow flow phenomenon-a new coronary microvascular disorder. General Cardiology Cardiology. 2002;97:197–202. Accessed October 22, 2022. https://www.karger.com. 10.1159/000063121 12145474

[40] Fearon WF, Farouque HMO, Balsam LB (2003). Comparison of coronary thermodilution and doppler velocity for assessing coronary flow reserve. Circulation.

[41] Singh M, Shah T, Khosla K (2012). Safety and efficacy of intracoronary adenosine administration in patients with acute myocardial infarction undergoing primary percutaneous coronary intervention: a meta-analysis of randomized controlled trials. Ther Adv Cardiovasc Dis.

[42] Tamis-Holland JE, Jneid H, Chair V (2019). On behalf of the American Heart Association Interventional Cardiovascular Care Committee of the Council on Clinical Cardiology; Council on Cardiovascular and Stroke Nursing; Council on Epidemiology and Prevention; and Council on Quality of Care and Outcomes Research Contemporary Diagnosis and Management of Patients With Myocardial Infarction in the Absence of Obstructive Coronary Artery Disease Circulation. Circulation.

[43] Marrone A, Pavasini R, Scollo E (2022). Acetylcholine use in modern cardiac catheterization laboratories: a systematic review. J Clin Med.

[44] Kaski JC (2018). Provocative tests for coronary artery spasm in MINOCA: necessary and safe?. Eur Heart J.

[45] Ong P, Athanasiadis A, Borgulya G (2014). Clinical usefulness, angiographic characteristics, and safety evaluation of intracoronary acetylcholine provocation testing among 921 consecutive white patients with unobstructed coronary arteries. Circulation.

[46] Ong P, Athanasiadis A, Hill S, Vogelsberg H, Voehringer M, Sechtem U. Coronary artery spasm as a frequent cause of acute coronary syndrome. The CASPAR (Coronary Artery Spasm in Patients with Acute Coronary Syndrome) study. J Am Coll Cardiol. 2008;52(7):523–527. 10.1016/J.JACC.2008.04.050. 10.1016/j.jacc.2008.04.050 18687244

[47] Montone RA, Rinaldi R, del Buono MG, et al. Safety and prognostic relevance of acetylcholine testing in patients with stable myocardial ischaemia or myocardial infarction and non-obstructive coronary arteries. EuroIntervention*.* 2022;18(8). 10.4244/EIJ-D-21-00971. 10.4244/EIJ-D-21-00971 PMC10241282 35377315

[48] Ong P, Athanasiadis A, Borgulya G (2014). Clinical usefulness, angiographic characteristics, and safety evaluation of intracoronary acetylcholine provocation testing among 921 consecutive white patients with unobstructed coronary arteries. Circulation.

[49] Godo S, Suda A, Takahashi J, Yasuda S, Shimokawa H (2021). Coronary microvascular dysfunction. Arterioscler Thromb Vasc Biol.

[50] Beltrame JF, Crea F, Kaski JC, et al. International standardization of diagnostic criteria for vasospastic angina. 10.1093/eurheartj/ehv351. 10.1093/eurheartj/ehv351 26245334

[51] Lacey MJ, Raza S, Rehman H, Puri R, Bhatt DL, Kalra A (2019). Coronary embolism: a systematic review. Published online.

[52] Kardasz I, de Caterina R. Myocardial infarction with normal coronary arteries: a conundrum with multiple aetiologies and variable prognosis: an update. 10.1111/j.1365-2796.2007.01788.x. 10.1111/j.1365-2796.2007.01788.x 17391108

[53] Pasupathy S, Air T, Dreyer RP, Tavella R, Beltrame JF (2015). Systematic review of patients presenting with suspected myocardial infarction and nonobstructive coronary arteries. Circulation.

[54] Lindahl B, Baron T, Erlinge D (2017). Medical therapy for secondary prevention and long-term outcome in patients with myocardial infarction with nonobstructive coronary artery disease. Circulation.

[55] He CJ, Zhu CY, Zhu YJ (2020). Effect of exercise-based cardiac rehabilitation on clinical outcomes in patients with myocardial infarction in the absence of obstructive coronary artery disease (MINOCA). Published online.

[56] Nordenskjöld AM, Agewall S, Atar D (2021). Randomized evaluation of beta blocker and ACE-inhibitor/angiotensin receptor blocker treatment in patients with myocardial infarction with non-obstructive coronary arteries (MINOCA-BAT): rationale and design: MINOCA-BAT: rationale and design. Am Heart J.

[57] Handberg EM, Merz CNB, Cooper-Dehoff RM (2021). Rationale and design of the Women’s Ischemia Trial to Reduce Events in Nonobstructive CAD (WARRIOR) trial. Am Heart J.

[58] Amsterdam EA, Wenger NK, Brindis RG (2014). 2014 AHA/acc guideline for the management of patients with non-ST-elevation acute coronary syndromes: a report of the American College of Cardiology/American Heart Association Task Force on Practice Guidelines. J Am Coll Cardiol.

[59] Hung MJ, Cherng WJ, Cheng CW, Yang NI. Effect of antispastic agents (Calcium antagonists and/ or isosorbide dinitrate) on high-sensitivity C-reactive protein in patients with coronary vasospastic angina pectoris and no hemodynamically significant coronary artery disease. 10.1016/j.amjcard.2004.08.064. 10.1016/j.amjcard.2004.08.064 15619396

[60] Ford TJ, Rocchiccioli P, Good R, et al. Systemic microvascular dysfunction in microvascular and vasospastic angina. 10.1093/eurheartj/ehy576. 10.1093/eurheartj/ehy529 PMC6284165 30165438

[61] Beltrame JF, Crea F, Camici P (2009). Mini-symposium advances in coronary microvascular dysfunction. Heart Lung Circ.

[62] Lerman A, Burnett JC, Higano ST, McKinley LJ, Holmes DR (1998). Long-term L-arginine supplementation improves small-vessel coronary endothelial function in humans. Circulation.

[63] Kılıç S, Aydın G, Çoner A (2020). Original investigation 176 prevalence and clinical profile of patients with myocardial infarction with non-obstructive coronary arteries in Turkey (MINOCA-TR): a national multi-center, observational study. Published online.

[64] Suhrs HE, Michelsen MM, Prescott E. Treatment strategies in coronary microvascular dysfunction: a systematic review of interventional studies. Microcirculation. 2019;26(3):e12430. 10.1111/MICC.12430. 10.1111/micc.12430 29130567

[65] Saw J, Mancini GBJ, Humphries KH (2016). Contemporary review on spontaneous coronary artery dissection. J Am Coll Cardiol.

[66] Rogowski S, Maeder MT, Weilenmann D (2017). Spontaneous coronary artery dissection: angiographic follow-up and long-term clinical outcome in a predominantly medically treated population. Catheter Cardiovasc Interv.

[67] Rashid HNZ, Wong DTL, Wijesekera H (2016). Incidence and characterisation of spontaneous coronary artery dissection as a cause of acute coronary syndrome - a single-centre Australian experience. Int J Cardiol.

[68] Tweet MS, Olin JW, Bonikowske AR, Adlam D, Hayes SN (2021). Physical activity and exercise in patients with spontaneous coronary artery dissection and fibromuscular dysplasia ischaemic heart disease. Eur Heart J.

[69] Samuel R, Alfadhel M, McAlister C, Nestelberger T, Saw J. Cardiac rehabilitation following coronary artery dissection: recommendations and patient considerations. 2021;19(11):1005–1012. 10.1080/14779072.2021.2013812. 10.1080/14779072.2021.2013812 34965826

